# A Labour of Labs: Improving Confidence in Postpartum Physiology

**DOI:** 10.1192/bjo.2026.11513

**Published:** 2026-06-30

**Authors:** Boris Warszawski, Yasmine Jabbar

**Affiliations:** 1 South London and Maudsley NHS Foundation Trust, London, United Kingdom; 2 Royal Free London NHS Foundation Trust, London, United Kingdom

## Abstract

**Aims:**

Clinicians working in perinatal psychiatric settings frequently encounter blood results that fall outside standard reference ranges due to the physiological changes of the “fourth trimester”. While information regarding accepted blood test levels throughout pregnancy is readily available, there is a lack of comprehensive, concise guidance for interpreting postpartum blood parameters and the time they require to return to baseline. This can lead to diagnostic uncertainty, unnecessary repeat investigations, and inappropriate specialist referrals. This Quality Improvement Project (QIP) was developed to address this gap in clinical confidence and reduce the burden of unnecessary medical interventions in the postnatal period. The project aimed to develop a comprehensive, evidence-based reference tool for postpartum blood parameter recovery timeframes and to evaluate its impact on clinician confidence and decision-making.

**Methods:**

A reference guidance document was developed through review of published literature and clinical guidelines. This document summarized postpartum trends and accepted reference ranges for common investigations, including haematological, biochemical, and inflammatory markers (e.g., FBC, LFTs, U&Es, and thyroid function). To evaluate the impactof this intervention, an anonymous electronic survey was distributed to clinicians to assess their confidence levels before and after providing access to the guide. Improvement was judged based on clinicians’ self-reported confidence using a Likert scale, and qualitative analysis was performed on free-text feedback provided in the survey.

**Results:**

The survey was sent to 53 clinicians, with 25 responses received at the time of writing. Preliminary survey data demonstrated a marked increase in clinician confidence following the introduction of the reference guide. Participants reported greater clarity in distinguishing normal physiological postpartum shifts from true pathology. Early qualitative feedback suggests a reduced perceived need for specialist referrals and repeat blood tests, supporting the tool’s utility in streamlining clinical care. Data collection is ongoing.

**Conclusion:**

This QIP highlights a need for accessible, postnatal-specific guidance for doctors working in psychiatry. The resulting reference tool is a low-cost, sustainable intervention that improves clinician confidence and supports appropriate resource allocation. This guidance is considered for inclusion in local induction materials for doctors on psychiatric perinatal wards, with next steps including dissemination to wards nationally and potential for broader application in primary care.

